# Supplementary material for: Identification of Metabonomics Changes in Longissimus Dorsi Muscle of Finishing Pigs Following Heat Stress through LC-MS/MS-Based Metabonomics Method
Source: Animals (Basel). 2020 Jan 13;10(1):129. doi: 10.3390/ani10010129 (PMC7022765; doi:10.3390/ani10010129)
Supplement: Supplementary file 1 [file animals-10-00129-s001.pdf]

Supplementary material

Table S1. Composition and nutrient levels of the experimental diet (as-fed basis) %.

Items	Content
Ingredients	
Corn	66.20
Soybean meal (42.8% CP)	20.00
Wheat bran	6.50
Wheat middlings	4.00
Limestone	1.00
$CaHPO_4$	0.60
NaCl	0.40
Premix[1)]	1.00
L-Lys ·HCl	0.30
Total	100.00
Nutrient levels[2)]	
Digestible energy (MJ /kg)	13.39
Crude protein (%)	15.73
Calcium (%)	0.65
Total phosphorus (%)	0.41
Available phosphorus (%)	0.17
Lysine (%)	0.92
Met+Cys (%)	0.54

[1)] Premix provided the following per kg of the diet: vitamin A 2512 IU, vitamin D_3 1200 IU, vitamin E 34 IU, vitamin K_3 1.5 mg, vitamin B_{12} 17.6 μg, riboflavin 2.5 mg, pantothenic acid 6.8 mg, nicotinic acid 20.3 mg, choline 351 mg, Mn 10 mg, Fe 50 mg, Zn 50 mg, Cu 10 mg, I 0.3 mg, Se 0.3 mg. [2)] Digestible energy (DE) and available phosphorus (AP) were calculated values, while the others were measured values.

Table S2. Effects of constant heat stress on the growth performance in finishing pigs (n = 8).

Items	CR group	HS group	PF group	P-value
Average daily feed intake (ADFI)/g	2936^{Aa}	2449^{Bb}	2472^{Bb}	＜0.01
Average daily gain (ADG)/g	873^{a}	649^{b}	750^{ab}	0.04
Feed/ Gain Ratio (F/G)	0.296	0.264	0.303	0.27

In the same row，values with different small letter superscripts mean significant difference ($p < 0.05$) and with different capital letter superscripts mean significant difference ($p < 0.01$), while with the same or no letter superscripts mean no significant difference ($p < 0.05$) .

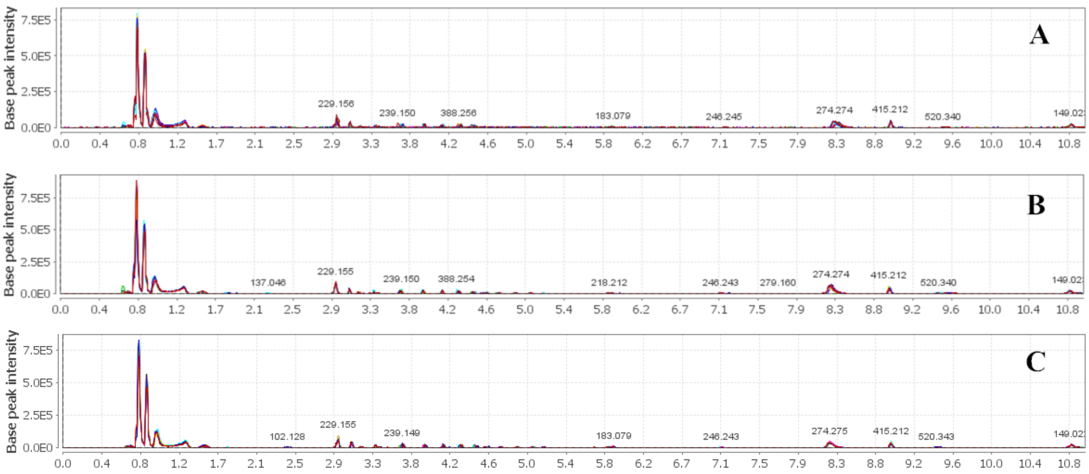

Figure S1. Base peak intensity (BPI) chromatograms of LD muscle samples using UPLC-ESI-QTOF-MS. A: BPI chromatograms of muscle samples obtained from CR pigs in positive mode; B: BPI chromatograms of muscle samples obtained from HS pigs in positive mode; C: BPI chromatograms of muscle samples obtained from PF pigs in positive mode.

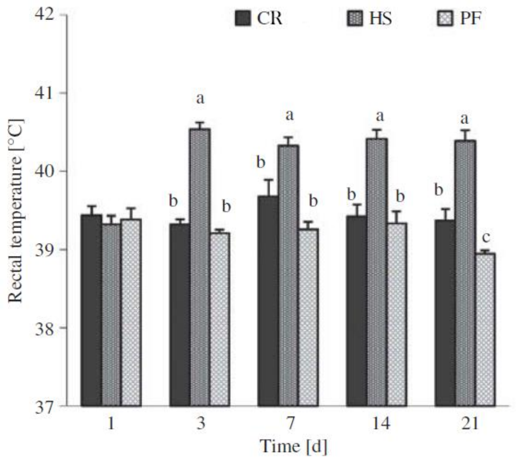

Figure S2. Effects of permanent heat exposure on rectal temperature.

Notes: CR, Control group housed at 22 ℃ with ad libitum feeding; HS, Group HS housed at 30 ℃ with ad libitum feeding; PF, Group PF housed at 22 ℃ and was pair-fed according to Group HS.

Values are means (n = 8) with their standard error, represented by vertical bars. Within time, alues with different letters (a, b, c) are significantly different ($p < 0.05$).
